# How to Get Pure Water

**Published:** 1880-01

**Authors:** 


### HOW TO GET PURE WATER.

In some sections of the country it is not possible to get good water except at great expense. In that case, rain water may be saved in cement cisterns or iron tanks, and kept pure for a long time with proper care ; but, where it is practicable, no water supply is so safe as that procured from "driven wells." But, after thousands of "driven wells" were constructed, a patent was granted—unexpectedly and as some think unjustly—the vital claim of which is for "a well, to the lining of which a pump is attached." In plain words, for a well made by driving down an iron pipe to the water, and screwing on a pump. For this privilege the patentee claims $10. Fortunately, there is an easy road around this obstruction ; and a far better well, in some respects, can be made outside this patent. If the soil is tolerably free from stones, a well can usually be made for a few dollars on the following plan : Get enough galvanized iron pipe, of one and a quarter-inch caliber, to reach the water. Have it cut in lengths convenient to drive—say 6 to 8 feet—and couplings to screw the pieces together. Then get a piece of plain iron tube, same size and 2 feet long. Have about 100 quarter-inch holes drilled through this piece of pipe, to admit the water and sand. Then have a blacksmith weld a piece of iron 6 inches long, and size of caliber, into one end of this short piece, leaving about 3 inches out, which he must hammer down to a point, not sharp, but blunt and strong; have him harden it so that it will split and penetrate quite large stones on its passage down. Screw on one length of pipe, and then, placing the point where you want the well, have one man hold a wooden beetle on the upper end of the pipe, while another man strikes the beetle with a heavy sledge hammer It will take from two hours to two days to drive down twenty feet of pipe, depending on the nature of the soil. With a small piece of iron or lead attached to a string, soundings for water can be made from time to time as the work proceeds. It is best to drive some two or three feet after water is reached, but not to go entirely through it. When the pipe is thus driven down, it is really an open well, it matters not if it be 1 inch in diameter or 6 feet, it is an open well nevertheless, and no man can contest your right to make such a well. Having now a well, get a three-quarter-inch galvanized pipe, attach a pump to it, and put it in the well. Pump out the sand, and you have a well into which impurities can not enter. By making the well of 1½ inch pipe, an inch pipe could be used with the pump; but I believe the well described of sufficient size to supply water for fifty families. In places where a "driven well" is impracticable, or where it is desirable to utilize an open well, either of the following plans may be adopted. Clean out the well, then get a galvanized iron pipe 1 inch in diameter and long enough to reach to within a foot of the bottom of well, and attach a pump to it. Plug up lower end of pipe with a piece of iron or hard wood. Drill 20 or 30 quarter-inch holes through the pipe near the lower end, to admit the water. Put a large stone in the center at bottom of well, on which to stand the pipe. Pack large round or "cobble" stones around the pipe, up 3 or 4 feet; then smaller stones, and next gravel and sand. Then pack in clay until the well is filled. This becomes practically a "driven well." The second plan is, after cleaning out the well, to line it with glazed earthen pipes. Put the pieces one on the other, packing outside with clay, and putting cement around the joints until the top is reached. This will exclude the surface water. Pipes of any diameter may be used—from 6 inches to 2 feet. If people would heed these suggestions, much sickness and suffering might be prevented.

